# A gene-by-sex interaction for nicotine reward: evidence from humanized mice and epidemiology

**DOI:** 10.1038/tp.2016.132

**Published:** 2016-07-26

**Authors:** R E Bernardi, K Zohsel, N Hirth, J Treutlein, M Heilig, M Laucht, R Spanagel, W H Sommer

**Affiliations:** 1 Institute of Psychopharmacology, Central Institute of Mental Health, Medical Faculty Mannheim/Heidelberg University, Mannheim, Germany; 2 Department of Child and Adolescent Psychiatry, Central Institute of Mental Health, Medical Faculty Mannheim/Heidelberg University, Mannheim, Germany; 3 Genetic Epidemiology, Central Institute of Mental Health, Medical Faculty Mannheim/Heidelberg University, Mannheim, Germany; 4 Center for Social and Affective Neuroscience, Linköping University, Linköping, Sweden; 5 Addiction Medicine, Central Institute of Mental Health, Medical Faculty Mannheim/Heidelberg University, Mannheim, Germany

## Abstract

It has been proposed that vulnerability to nicotine addiction is moderated by variation at the μ-opioid receptor locus (OPRM1), but results from human studies vary and prospective studies based on genotype are lacking. We have developed a humanized mouse model of the most common functional OPRM1 polymorphism rs1799971_A>G (A118G). Here we use this model system together with a cohort of German youth to examine the role of the OPRM1 A118G variation on nicotine reward. Nicotine reinforcement was examined in the humanized mouse model using i.v. self-administration. Male (*n*=17) and female (*n*=26) mice homozygous either for the major human A allele (AA) or the minor G allele (GG) underwent eight daily 2 h sessions of nicotine self-administration. Furthermore, male (*n*=104) and female (*n*=118) subjects homozygous for the A allele or carrying the G allele from the Mannheim Study of Children at Risk were evaluated for pleasurable and unpleasant experiences during their initial smoking experience. A significant sex-by-genotype effect was observed for nicotine self-administration. Male 118GG mice demonstrated higher nicotine intake than male 118AA mice, suggesting increased nicotine reinforcement. In contrast, there was no genotype effect in female mice. Human male G allele carriers reported increased pleasurable effects from their first smoking experience, as compared to male homozygous A, female G and female homozygous A allele carriers. The 118G allele appears to confer greater sensitivity to nicotine reinforcement in males, but not females.

## Introduction

Despite notable success in decreasing rates of smoking, tobacco use remains a major public health issue and the leading cause of preventable death worldwide.^1, 2^ The addictive properties of tobacco are largely attributable to nicotine,^3, 4^ and a resounding reminder of this is the rapid increase in the use of e-cigarettes, electronic delivery systems that enable long-term use of tobacco-free nicotine, which are increasingly popular among smokers. The ramifications of this recent development for tobacco control are a matter of intense debate.^5^ According to a recent US survey, e-cigarettes are increasing youth nicotine use,^6^ and may ultimately lead to smoking and nicotine addiction.

The individual response to nicotine varies widely, partly due to genetic factors. Moderate heritability (*h*^*2*^ ~0.6) has been found for nicotine addiction in large twin studies, and similar findings have also been obtained for initiation and use.^7, 8, 9^ Part of this risk has consistently been associated with variants of nicotinic acetylcholine receptor genes,^10, 11, 12^ as well as genes more directly involved in reward processing, such as those related to dopamine and opioid systems.^12, 13, 14, 15, 16, 17^ Understanding the role of genetic factors may allow for the development of personalized approaches to the prevention and treatment of nicotine addiction.

The rewarding properties of nicotine are mediated in part by μ-opioid receptors (MOR) encoded by the OPRM1 locus.^3, 18^ The reinforcing effects of nicotine are attenuated in mice lacking MOR^19, 20^ and MOR antagonists suppress nicotine self-administration in rats.^21, 22^ In humans, cigarette smoking increases the release of the endogenous MOR ligand β-endorphin,^23^ while naloxone, a MOR antagonist, decreases nicotine reward.^24^ Also, MOR availability in reward-related brain regions correlates with nicotine dependence and reward.^25, 26^

A single nucleotide polymorphism (SNP), *rs1799971:A>G* (A118G), exists within exon 1 of the OPRM1 gene and encodes a non-synonomous substitution (Asn40Asp) in the extracellular N-terminal loop of MOR, resulting in loss of a glycosylation site.^27, 28^ The precise molecular consequences of this polymorphism for nicotine reward remain unclear. Several studies have implicated the A118G variation with individual differences in nicotine reinforcement or addiction, but its role remains unclear and conflicting findings have been reported.

For instance, adolescents carrying the G allele were more likely to report 'liking' an initial smoking exposure than their AA counterparts.^29^ Another study in smokers reported reduced nicotine reinforcement in female G allele carriers, relative to AA homozygotes, but no genotype related effects in males.^30^ However, no effects of A118G genotype or sex on nicotine responses were found in nonsmokers that received nicotine via nasal spray.^31^ In a positron emission tomography study, male smokers carrying the G allele showed increased DA release in response to cigarette smoking in reward-related brain areas compared with AA subjects.^32^

It thus remains unclear whether OPRM1 A118G alters the susceptibility to smoking behaviors. This is in contrast to studies in alcohol research, in which findings from animal models, human laboratory studies and some, but not all, clinical trials indicate that the OPRM1 118G allele confers increased alcohol reward and an enhanced therapeutic response to naltrexone.^33, 34, 35, 36, 37, 38^ Further studies, both clinical and preclinical, are clearly necessary to clarify the pharmacogenetic role of this variant, which is of particular interest for individuals of European or Asian ancestry, where its frequency ranges from 15 to 50%.^39, 40^

Human genetic studies aimed at identifying risk variants suffer from two important potential confounds: linkage disequilibrium with other variants and stratification bias. To overcome this problem, we recently generated humanized mouse lines in which the endogenous mouse *Oprm1* exon 1 was replaced with the corresponding human sequences encoding either the A- or G allele of rs1799971.^38^ By differing only in a single nucleotide, the h/mOPRM1-118AA and h/mOPRM1-118GG mouse lines allow for examination of the functional consequences of each variant in isolation. 118GG mice replicate important phenotypes associated with the G allele in humans, including increased alcohol reward and sensitivity to the effects of opioid antagonist treatment on alcohol intake, as well as a decreased analgesic efficacy of morphine,^37, 38, 41^ thus validating these humanized mouse lines as a reverse-translational tool.

Here we used this humanized OPRM1 mouse model in a translational approach to clarify some of the reported genotype and sex discrepancies associated with the A118G variation as related to nicotine reinforcement. First, we ascertained differences in nicotine self-administration of 118AA and 118GG mice of both sexes. We then examined whether the mouse findings would translate to humans by evaluating the pleasurable and unpleasant effects of the first smoking experience reported by male and female participants of the Mannheim Study of Children at Risk, a prospective longitudinal study following infants from birth to young adulthood.^42, 43^

## Materials and methods

### Animal study

#### Animals

Adult C57Bl/6N (Charles River, Sulzfeld, Germany), and male and female AA and GG mice aged 12–16 weeks were single-housed in a temperature-controlled (21 °C) environment maintained on a 12 h light–dark cycle (lights on at 0600 hours). Food and water was available ad libitum. All experiments were performed in accordance with EU guidelines on the care and use of laboratory animals and were approved by the local animal care committee (Regierungspräsidium, Karlsruhe, Germany).

The generation of the h/m*OPRM1*-118AA and -118GG mice has been described previously.^38^ Briefly, two humanized mouse lines were generated on a C57BL/6 background. The mouse *Oprm1* exon 1 was replaced by the human sequence. One line, h/m*OPRM1*-118AA, was homozygous for the major human 118A allele. For h/mOPRM1-118GG, the same insert was used, but site-directed mutagenesis was first used to introduce a G in position 118. The lines are genetically identical, with the exception of the A→G substitution. The two lines were crossed and maintained through heterozygous breeding as a line carrying both alleles at the *OPRM1*-A118G site.

#### Drugs

**Figure 1:**
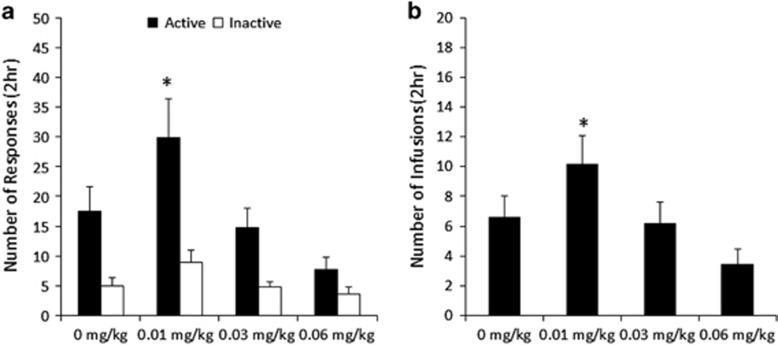
Nicotine dose–response in male C57Bl/6N mice. Male mice demonstrated increased responding (**a**) and an increased number of infusions obtained (**b**) for the 0.01 mg per kg dose nicotine relative to all other doses. *P<0.05 relative to other doses.

Nicotine hydrogen tartrate salt (Sigma-Aldrich, Steinheim, Germany) was dissolved in physiological saline (0.9% NaCl) for i.v. injection of 0, 0.01, 0.03 and 0.06 mg kg^−1^ per 35 μl infusion for nicotine dose–response testing and 0.01 mg kg^−1^ per 35 μl infusion for OPRM1 A118G characterization based on free base weight (final solution adjusted to ~pH 7 using NaOH). The 0.01 mg kg^−1^ per infusion dose has previously been shown to support self-administration in mice,^44, 45^ and in our hands results in the most robust responding and intake relative to other commonly-used doses (see ref. 46 and Figure 1).

#### Apparatus and behavioral procedures

Nicotine self-administration was assessed in 12 operant chambers (Med Associates, Fairfax, VT, USA) housed in light- and sound-attenuating cubicles. Each chamber (24.1 × 20.3 × 18.4 cm) was equipped with two levers (left and right), a food dispenser and a drug delivery system connected via infusion pump (PHM-100, Med Associates) located outside the cubicle. Operant chambers were controlled using Med-PC IV (Med Associates) software. Mice first underwent lever training under an Fixed Ratio 1 (FR1) schedule with 14 mg sweetened food pellets (TestDiet, St. Louis, MO, USA), as previously described.^46^ Following lever training, mice were implanted with an indwelling i.v. catheter (made in-house) into the jugular vein. Catheter patency was maintained with 0.15 ml heparanized saline (100 i.u. ml^−1^) containing Baytril (0.7 mg ml^−1^) administered daily throughout the experiment. After a 3 day recovery period, mice underwent daily 2 h nicotine self-administration for 8 consecutive days. Nicotine delivery was contingent on pressing on the active lever under an FR2 (two presses results in one reinforcer) schedule of reinforcement and paired with the 20 s  presentation of a blinking light stimulus (conditioned stimulus (CS)), which also served as a timeout period, during which lever presses were not reinforced. For all experiments, presses on the inactive lever were recorded but had no scheduled consequence. All behavioral testing was conducted during the light phase.

We performed a nicotine dose–response to confirm our use of the 0.01 mg kg^−1^ per infusion dose. Following nicotine self-administration (0.01 mg kg^−1^ per infusion) for 8 days as described above, male C57Bl/6N (*n*=7) animals were subjected to each of three doses (0, 0.03 and 0.06 mg kg^−1^ per infusion) during a single 2 h session on consecutive days. In addition, we evaluated nicotine self-administration (0.01 mg kg^−1^ per infusion) in male and female mice AA and GG mice (*n*=8–15 per group) during daily 2 h nicotine self-administration for 8 consecutive days. Based on the findings from this experiment, we then examined cue responding in male AA and GG mice (*n*=7–8 per group), during which self-administration procedures were assessed as described above, except that the mice were not subjected to catheter implantation or nicotine infusions, and thus were only assessed for responding for the blinking light CS.

### Human study

#### Participants

Subjects were participants of the Mannheim Study of Children at Risk, a prospective longitudinal study following infants at risk for later developmental disorders from birth to young adulthood, as previously described.^42, 43^ Children were primarily of Caucasian ethnicity (99%). From a total of 384 infants, 312 subjects participated in the 23-year assessment. About 225 reported to have ever smoked at least 1 cigarette (72.1%) and 3 had incomplete data, leaving 222 young adults (104 males, 118 females) to be included into the analyses for the present study. The study design was approved by the Ethics Committee of the University of Heidelberg. All participants provided written informed consent.

#### Measures

At the ages of 15, 19, 22 and 23 years, participants completed a detailed smoking inventory including age of smoking onset and lifetime tobacco use (for example, the presence of at least one period of daily smoking). This inventory is part of the Substance Use Questionnaire designed by Müller and Abbet^47^ in collaboration with the World Health Organization (for more details see ref. 48). To measure the individual's response to the initial experimentation with cigarettes, the early smoking experiences questionnaire^49^ was administered and the participants were asked to give global ratings of their pleasurable and unpleasant feelings (from 1=none to 4=intense) the first time they tried cigarettes. To reduce recall bias, the response to initial exposure was recorded at the assessment following smoking initiation (for example, smoking initiation at age 14 years: ratings at the assessment at age 15 years; smoking initiation at age 18 years: ratings at the assessment at age 19 years and so on). Smoking status at age 23 years and mean early smoking experiences are presented in Table 1. Psychosocial adversity as a potential confound was assessed 3 months after birth by rating the presence of 11 adverse family factors.^42^

#### Genotyping

Genomic DNA was extracted either from ethylenediaminetetraacetic acid anticoagulated venous blood or saliva according to standard procedures. The rs1799971 (or A118G) SNP of the OPRM1 gene was genotyped on a 7900HT Fast Real-Time PCR System (Life Technologies, Ober-Olm, Germany), using a TaqMan 5ʹ nuclease assay (TaqMan SNP Genotyping Assay ID C_8950074_1; Life Technologies). Three participants were homozygous and 62 heterozygous carriers of the G allele. About 157 participants were homozygous for the A allele, with no significant differences between males and females (*Χ*^2^=2.71, *P*=0.258). Genotype distribution did not significantly deviate from the Hardy–Weinberg equilibrium, neither in the entire sample (*P*=0.253) nor separately for males and females. Because of the low frequency of the G allele, homo- and heterozygous G allele carriers were combined to maximize the power of analyses.

### Statistical analysis

All statistical analyses were performed using SPSS (IBM, Armonk, NY, USA). For animal data, a repeated measures analysis of variance (ANOVA) was used to evaluate lever pressing and nicotine reinforcers during nicotine dose–response testing. For nicotine self-administration in AA and GG mice, ANOVAs were used to evaluate sex and genotype effects on lever pressing and nicotine reinforcers achieved across daily self-administration sessions; due to the residual effects of food training, the first 2 days of nicotine self-administration were omitted in all analyses. For human data, ANOVAs were used to evaluate pleasurable and unpleasant early smoking sensations as a function of sex and genotype. In terms of pleasant sensations, an ordinal interaction pattern was observed with a postulated lack of power in the traditional ANOVA approach.^50^ Thus, using the procedure suggested by Strube and Bobko^51^ and Elias and Cropanzano,^52^ we compared the means in male homozygous A allele carriers, female G and female homozygous A allele carriers. In the case of nonsignificant differences, groups were combined and tested against male G allele carriers. For progression to daily smoking, logistic regression analyses were calculated to examine the effects of the factors OPRM1 genotype (AA coded as 0, G coded as 1) and sex (male coded as 0, female as 1) and their interaction. All models included age of initial exposure to nicotine and psychosocial adversity as covariates. Significance was set at *P*<0.05.

## Results

### Nicotine dose response

Figure 1 shows the responding (a) and infusions obtained (b) for each nicotine dose. The 0.01 mg per kg dose refers to the final day of 8 days of self-administration prior to dose changes. A repeated measures ANOVA of dose × lever for active and inactive lever pressing revealed significant effects of lever (F(1,6)=15.0, *P*<0.05) and dose (F(3,18)=14.0, *P*<0.0005), and a significant lever × dose interaction (F(3,18)=8.0, *P*<0.005). Paired *t*-tests revealed that active lever pressing was greater in the 0.01 mg per kg dose than all other doses (all *t*(6)>2.9, *P*<0.05), with no difference in active lever pressing between the 0 and 0.03 mg kg^−1^ (*t*(6)=0.9, *P*>0.05) doses, and both of these doses greater than 0.06 mg kg^−1^ (all *t*(6)>3.3, *P*<0.05). Inactive lever pressing differed between the 0.01 mg per kg dose and both other nicotine doses (all *t*(6)>2.6, *P*<0.05), with no other significant differences among the different doses.

For infusions obtained, a repeated measures ANOVA of dose revealed a significant effect (F(3,18)=10.3, *P*<0.0005). Paired *t*-tests demonstrated that the number of infusions obtained for the 0.01 mg per kg dose was significantly higher than all other doses (all *t*(6)>2.7, *P*<0.05), with no difference in infusions between the 0 and 0.03 mg kg^−1^ (*t*(6)=0.3, *P*>0.05) doses, and both of these doses >0.06 mg kg^−1^ (all *t*(6)>2.7, *P*<0.05). These data demonstrate that the 0.01 mg per kg dose nicotine results in increased responding and infusions obtained relative to other doses of nicotine.

### Increased nicotine self-administration in male h/mOPRM1-118GG mice

**Figure 2:**
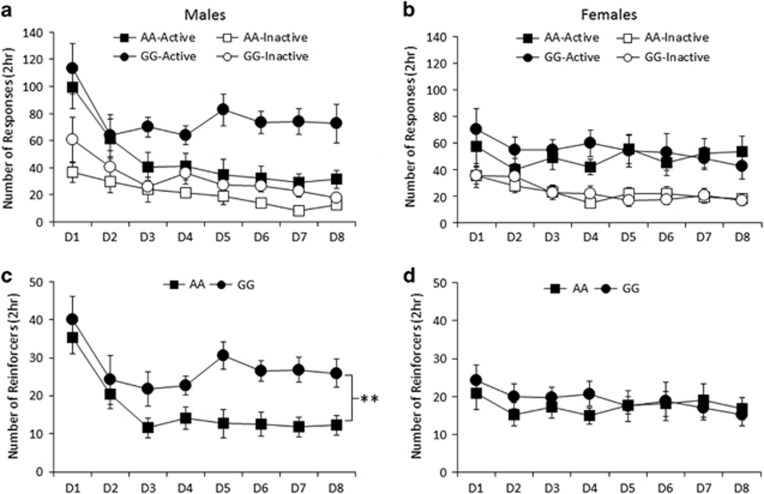
Self-administration of nicotine in male and female AA and GG mice. (**a** and **b**) Both male and female AA and GG mice showed discrimination between responding on the active and inactive levers (**a** and **b**). Data represent mean (±s.e.m.) number of presses on the active/inactive levers during eight daily 2 h sessions of nicotine self-administration (0.01 mg kg^−1^ per infusion). (**c**) Male GG mice showed increased nicotine intake relative to AA male mice, while (**d**) female AA and GG mice did not differ in nicotine reinforcers achieved. Data represent mean (±s.e.m.) number of reinforcers achieved during 8 daily 2 h sessions of nicotine self-administration (0.01 mg kg^−1^ per infusion). **P<0.005.

I.v. self-administration is an established operant method to assess the reinforcing properties of nicotine. All mice (AA: 9 male, 15 female mice, GG: 8 males, 11 females) rapidly acquired stable responding for nicotine and learned to discriminate the active versus the inactive lever during 8 daily 2 h sessions under an FR2 schedule (2 presses=1 nicotine infusion) of reinforcement (Figures 2a and b for males and females, respectively), indicated on days 3–8 by a significant main effect of lever (F(1,39)=92.0, *P*<0.001) (ANOVA: lever × day × sex × genotype) and significant *post hoc* paired *t*-tests (active vs inactive lever) for all groups (all *t*>4.1, *P*<0.003). Furthermore, male GG mice demonstrated significantly higher active lever presses than AA mice (*t*(15)=3.6, *P*<0.005), but no difference in inactive lever presses (*t*(15)=1.8, *P*>0.05), while female AA and GG mice did not differ on either active (*t*(24)=0.2, *P*>0.05) or inactive (*t*(24)=0.1, *P*>0.05) lever pressing.

For nicotine reinforcers obtained (Figures 2c and d for males and females, respectively), a three-way ANOVA of days 3–8 (day × sex × genotype) revealed a significant genotype × sex interaction (F(1,39)=4.8, *P*<0.05) and main effect of genotype (F(1,39)=6.1, *P*<0.05), but no other significant effects (all F<1, except day × sex × genotype: F(5, 195)=1.9, *P*>0.05). For male mice, a two-way ANOVA (day × genotype) of nicotine reinforcers obtained on days 3–8 revealed a significant main effect of genotype (F(1,15)=13.3, *P*<0.005), but no other significant effects (all F(5,75)<1.4, *P*>0.05), indicating that male 118GG mice self-administered significantly more nicotine than male 118AA mice. For female mice, a two-way ANOVA (day × genotype) of nicotine reinforcers achieved on days 3–8 revealed no significant effects (all F<1), indicating no genotype-specific differences among female mice. These data demonstrate that nicotine self-administration in the humanized mouse lines differ as a function of OPRM1 A118G genotype and sex and suggest greater nicotine reinforcement in male h/mOPRM1-118GG mice.

### No difference in cue responding in male h/mOPRM1-118GG mice

**Figure 3:**
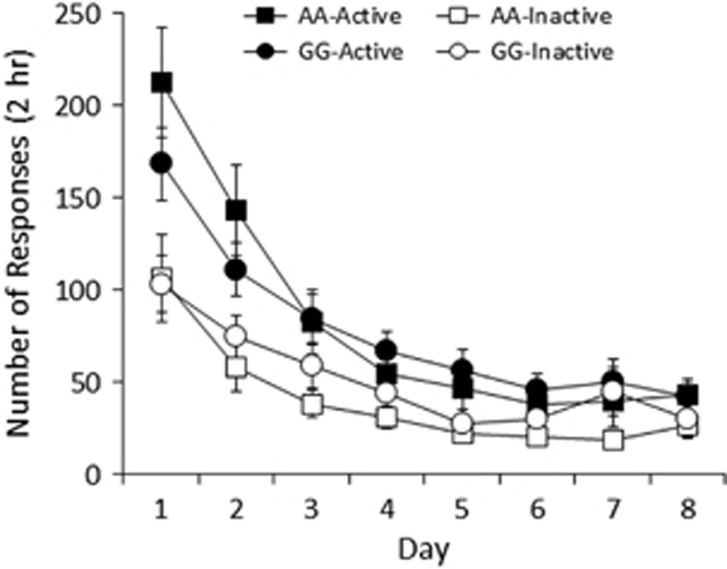
Cue responding in male AA and GG mice. AA and GG mice showed similar responding for the blinking light CS. Data represent mean (±s.e.m.) number of presses on the active/inactive levers during eight daily 2 h sessions. CS, conditioned stimulus.

Figure 3 shows the responding for the blinking light CS. A three-way ANOVA of days 3–8 (lever × genotype × day) revealed significant main effects of lever (F(1,13)=13.3, *P*<0.005) and day (F(1.7,22.5)=5.3, *P*<0.05), and a significant lever × day interaction (F(5,65)=3.0, *P*<0.05), but no other significant effects (all Fs<1, except genotype: F(1,13)=1.3, *P*=0.27). These results indicate no genotype differences in sensory reinforcement, and a progressive decline in active relative to inactive lever responding in the absence of an unconditioned stimulus, and thus suggest that the differences in nicotine self-administration demonstrated in male AA and GG mice are likely due to differences in nicotine reward and not differences in sensory reinforcement.^44, 53^

### Increased pleasurable initial smoking experience in male G allele carriers

**Figure 4:**
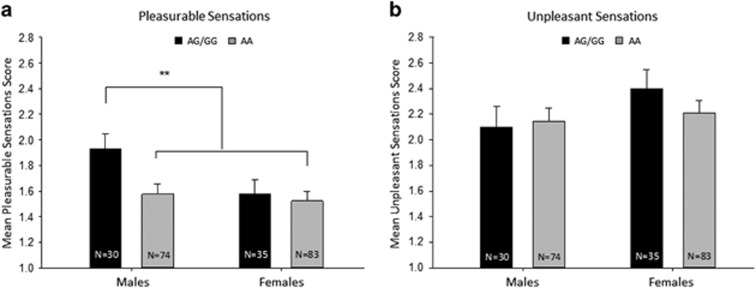
Early (**a**) pleasurable and (**b**) unpleasant sensations of initial smoking experience, grouped by sex and OPRM1 genotype. Male G allele carriers identified an initial smoking experience as more pleasurable than male homozygous A allele carriers, female G and female homozygous A allele carriers (**a**), with no differences in unpleasant sensations between the groups (**b**). Data represent mean (±s.e.m.) scores on the Early Smoking Experiences questionnaire adjusted by the inclusion of age of initial exposure to nicotine and psychosocial adversity as covariates. **P<0.005.

Human male G allele carriers rated their initial smoking experience as more pleasurable than male homozygous A allele carriers, female G allele carriers, and female homozygous A allele carriers. Figures 4a and b show the mean (±s.e.m.) scores on the Early Smoking Experiences questionnaire for pleasurable and unpleasant smoking experiences, respectively, for each genotype group, adjusted for age at initial exposure to nicotine and psychosocial adversity. A two-way ANOVA (sex × genotype) revealed main effects of sex (F(1,216)=4.37, *P*<0.05) and genotype (F(1,216)=4.28, *P*<0.05), but no significant interaction (F(1,216)=2.35, *P*>0.05). Mean pleasurable sensations did not differ between male homozygous A allele carriers, female G and female homozygous A allele carriers (Fs<1). When combined, a highly significant difference occurred in contrast to male carriers of the G allele (F(1,218)=8.48, *P*<0.005). For unpleasant early smoking sensations, a two-way ANOVA (sex × genotype) revealed no significant main effects of sex (F(1,216)=1.91, *P*>0.05) or genotype (F(1,216)=0.31, *P*>0.05) and no significant interaction (F(1,216)=0.77, *P*>0.05). Furthermore, there were no significant main effects of sex (odds ratio (OR)=1.40, 95% confidence interval (CI)=0.69–2.85, *P*=0.354) or genotype (OR=2.28, CI=0.91–5.70, *P*=0.078) or sex × genotype interaction (OR=1.66, CI=0.47–5.85, *P*=0.431) with regard to progression to daily smoking until age 23 years.

## Discussion

The most salient finding of the present translational study is a striking similarity between species in the sensitivity to nicotine reinforcement as a function of genotype and sex. Using a humanized mouse model, we isolated the influence of the OPRM1 A118G variation from potential confounds commonly present in human studies and identified a sex-specific influence of the G allele on nicotine self-administration, which then provided a basis for investigation of reported initial smoking experiences in a human population sample, in which we further demonstrated that male carriers of the 118G allele showed higher initial rewarding effects of nicotine compared with male 118A homozygous and females regardless of genotype. The convergent genetic findings obtained using this translational strategy support a role for the 118G allele as a key predictor of increased nicotine reward in males but not females.

Reports of the association of the A118G polymorphism with smoking behaviors are inconsistent.^29, 30, 31, 54, 55, 56, 57, 58^ In addition to the present study, the G allele has been associated with increased pleasurable effects during an initial smoking exposure among adolescents^29^ and higher tobacco use in some adult populations.^54, 55^ Furthermore, higher nicotine-evoked striatal dopamine release was found in male smokers carrying the G allele by positron emission tomography using [(11)C]raclopride (32). The latter finding is consistent with observations of selectively enhanced alcohol stimulation and reward in male, but not female rhesus macaques carrying a orthologous OPRM1 allele,^59^ and with a human positron emission tomography study demonstrating enhanced dopamine release following an alcohol challenge in male G allele carriers.^38^ These observations in turn are paralleled by experiments in the humanized mouse lines, in which male GG mice have shown a markedly enhanced alcohol-evoked dopamine release in the ventral striatum,^38^ as well as increased behavioral measures of alcohol reinforcement^37^ relative to male AA mice. Importantly, with the exception of the non-human primate study, females have not been characterized in these studies.

In addition, in another mouse model of the A118G polymorphism, in which an orthologous SNP (A112G) was introduced into the murine exon 1, resulting in a functionally similar amino acid substitution, male and female GG mice showed greater acute heroin-induced dopamine levels in the striatum and greater heroin self-administration relative to their AA conspecifics.^60^ Interestingly, male, but not female GG mice, demonstrated a greater escalation of heroin intake during extended access sessions, relative to their sex-specific AA mice.^60^ Together these data suggest greater reinforcement across a number of drugs of abuse in males carrying the G allele. Although our data are consistent with these previous findings demonstrating enhanced drug intake in mice homozygous for the G allele, an alternative hypothesis that must be considered is that the OPRM1 polymorphism results in a reduction in the putative anxiogenic effects of acute and chronic drug administration. Many previous studies have demonstrated anxiety-like behaviors associated with nicotine withdrawal (reviewed in ref. 61), which can be modulated by MOR action,^62^ and thus may be differentially mediated by the OPRM1 polymorphism. Further studies are needed to clarify this issue.

Clinical studies have identified differences in smoking-related behaviors between men and women,^63, 64, 65^ including increased sensitivity to the rewarding effects of nicotine in women.^64, 66, 67^ Preclinical studies have also identified sex differences in nicotine reinforcement, with female rodents demonstrating faster acquisition of self-administration at lower doses of nicotine^68^ and higher magnitude of nicotine conditioned place preference^69, 70^ than males. Increased sensitivity to the rewarding effects of drugs of abuse in females have extensively been attributed to estrogen.^71^ Estrogen increases dopamine release in response to drugs of abuse, including nicotine, and alters the striatal dopamine D1/D2 receptor balance towards stronger activation of medium spiny neurons by dopamine, a mechanism that is important for associative, as well as motor learning.^72^ Furthermore, estrogen alters the density and binding characteristics of MOR in the brain.^73, 74, 75^ Under the present experimental conditions, however, we did not observe higher responding for nicotine in female mice compared with males, although we did not control for estrus cycle. A differential effect of estrogen on the density and binding characteristics of MOR in AA and GG carriers is one potential explanation for our findings. Estrogen levels are low in females pre menarche. In our human sample, 23 females reported an initial smoking experience prior to menarche. Only three G allele carriers were found in this subgroup, but interestingly, these subjects recounted higher pleasurable sensations from their initial smoking experience than A homozygous females. Thus, further studies evaluating the response to nicotine in GG and AA female mice, as well as in humans, are warranted.

Enhanced drug reward in OPRM1 118G carriers could potentially increase the risk for excessive use and the development of substance use disorders, including smoking addiction, although this notion is not supported by human epidemiological data. A meta-analysis of available association studies failed to detect evidence for an effect of the A118G polymorphism on the risk for nicotine dependence.^76^ Also, in the cohort of youth analyzed here, we did not find evidence of a moderating effect of genotype on progression to daily smoking. Our findings contrast those from Kleinjan *et al.*,^77^ who reported that in males aged 13–15 years, the A allele was associated with a faster development of smoking behavior, whereas in females, G allele carriers showed a faster development of smoking. Further studies on adolescent smoking should address the role of genetic variation at the MOR gene locus on smoking behavior.

One of the primary implications of our findings is that similar to the treatment of alcoholism, MOR antagonists may be useful for smoking cessation and the efficacy of such treatments may be determined by pharmacogenetic effects of the A118G polymorphism. Naltrexone and nalmefene are both clinically-approved for the treatment of alcohol use disorders, and have a demonstrated efficacy in reducing consumption in large meta-analyses including more than 10 000 patients.^33, 78, 79, 80^ For naltrexone, the pharmacogenetic influence of the A118G variant on treatment response has been established by meta-analysis.^33^ This was recently supported by the demonstration of increased sensitivity to both nalmefene and naltrexone treatment in OPRM1 118G mice,^37^ indicating that treatment with MOR antagonists may have a larger effect size when targeted to patients with a genotype that predicts response.

In contrast to alcohol studies, clinical trials examining the effectiveness of MOR antagonists in smoking cessation have been inconsistent. Although naltrexone has been demonstrated to decrease nicotine reward, smoking urge and cigarette consumption in various paradigms, and increase the efficacy of nicotine-replacement therapies for smoking cessation,^24, 81, 82, 83, 84, 85^ consistent with a role for MOR in nicotine reinforcement, several other studies have demonstrated little or no success with MOR antagonists. In fact, recent meta-analyses of the effect of MOR antagonists on smoking cessation concluded that naltrexone had no beneficial effect on abstinence from smoking.^86, 87^ To date, only one study has examined the efficacy of MOR antagonists as a function of OPRM1 A118G genotype regarding smoking behaviors.^30^ No effect of naltrexone as a function of either genotype or sex was found on the reinforcing value of nicotine. Interestingly, in this study, female carriers of the G allele demonstrated a reduced relative reinforcing value of nicotine as compared with female AA carriers under basal conditions, with no differences in genotype among male subjects, contrasting our rodent and human findings. Nonetheless, the significance of our findings with respect to MOR-directed pharmacotherapies remains to be determined.

The present study has several limitations. First, the experiments did not elucidate the molecular mechanism underlying increased nicotine reward in male 118G carriers. Initially it was believed that the 118G variant conferred increased MOR affinity for the endogenous ligand β-endorphin,^57^ but dependent on experimental conditions, the G allele may act as gain-of-function or loss-of-function variant.^28, 88, 89, 90^ Increased receptor affinity was not observed in the humanized mouse lines used here,^38^ but in the A112G mouse model^60^ male GG mice showed increased functional output in reward-related brain areas compared with AA males on stimulation with the MOR-selective agonist [D-Ala2, N-MePhe4, Gly-ol]-enkephalin (DAMGO).^91^ MOR within striatal regions may also mediate the effects of the polymorphism on nicotine reinforcement in the humanized mice. However, drug-taking and drug-seeking require concerted activity between a number of subcortical and cortical regions. The insular cortex has been demonstrated to be a key mediator of many of the effects of nicotine and other drugs of abuse.^92, 93^ For example, damage to the insula diminishes tobacco smoking in humans,^94, 95^ and in alcohol-dependent subjects G allele carriers of the OPRM1 polymorphism show greater insular activation in response to alcohol cues.^96^ And while the elucidation of brain region and sex-specific effects mediated by the receptor mutations require further research, it is also possible that the G-variant may act indirectly on reward mechanisms by enhancing beta-endorphin release. However, *in vivo* measurements of nicotine-evoked beta-endorphin release have thus far been proven difficult.^25, 97, 98^ A second limitation is that because of the complexity of the experiments, we only tested homozygotic mice, while in populations of European ancestry, including our youth cohort, GG-homozygotes are rare, and the observed effects are driven by heterozygote G allele carriers. Thus, we cannot determine whether the effects of the 118G allele on nicotine reward are dominant or co-dominant. Finally, because the human sample was comprised of children at risk, the ability to generalize to the general population may be limited.

In conclusion, we report here convergent genetic evidence for a sex-by-genotype interaction on nicotine reward mediated by the most common functional variation at the OPRM1 locus. Our translational approach of using a humanized mouse model for this polymorphism is sensitive and specific for identifying genotype-dependent phenotypic responses that can be utilized for testing in human populations. Our demonstration of greater sensitivity to the rewarding effects of nicotine in males carrying the OPRM1 118G allele is important for the development of personalized approaches to the prevention and treatment of smoking addiction.

## Figures and Tables

**Table 1:** Smoking status at age 23 years and early smoking experiences in the epidemiological sample

Current monthly smoking: *n* (%)	132 (59.5)
Current daily smoking: *n* (%)	95 (42.8)
At least one period of daily smoking: *n* (%)	141 (63.5)
Age at first cigarette: *M* (s.d.; range)	13.9 (2.3; 8.0–21.7)
Pleasurable sensations: *M* (s.d.)	1.6 (0.7)
Unpleasant sensations: *M* (s.d.)	2.2 (0.9)
